# Prevalence of Functional Disabilities and Associations Among Disabilities, Violence, and HIV Among Adolescents and Young Adults in Lesotho

**DOI:** 10.1007/s44197-023-00184-3

**Published:** 2024-03-18

**Authors:** Greta M. Massetti, Caroline Stamatakis, Sana Charania, Francis B. Annor, Catherine E. Rice, Jennifer Hegle, Puleng Ramphalla, Masechache Sechache, Mookho Motheo

**Affiliations:** 1 Division of Violence Prevention, National Center for Injury Prevention and Control, Centers for Disease Control and Prevention, 4770 Buford Highway NE, Atlanta, GA 30341 USA; 2 Divison of Human Development and Disability, National Center on Birth Defects and Developmental Disabilities, Atlanta, USA; 3 Division of Global HIV and TB, Global Health Center, Centers for Disease Control and Prevention, Atlanta, USA; 4 Division of Global HIV and TB, Centers for Disease Control and Prevention, Maseru, Lesotho; 5 U.S. Agency for International Development, Maseru, Lesotho; 6 Ministry of Social Development, Government of Lesotho, Maseru, Lesotho

**Keywords:** HIV, Disabilities, Adolescents and young adults

## Abstract

**Introduction:**

Lesotho has the second-highest prevalence of HIV. Despite progress in achieving HIV epidemic control targets, inequities persist among certain groups, particularly associations between disability, HIV, and violence. We assessed the prevalence of disability and examined associations between disability and HIV and violence using data from the 2018 Lesotho Violence Against Children and Youth Survey (VACS).

**Methods:**

Lesotho VACS was a nationally representative survey of females and males ages 13–24. We assessed the associations between disability status and HIV, sexual risk behaviours, and violence using logistic regression, incorporating survey weights.

**Results:**

Weighted functional disability prevalence was 14.1% for females (95% confidence interval [CI] 12.7–15.4) and 7.3% for males (5.3–9.2). Compared with females with no disabilities, females with disabilities had higher odds of being HIV positive (adjusted odds ratio [aOR] 1.92, 1.34–2.76), having transactional sex (aOR 1.79, 1.09–2.95), and experiencing any lifetime violence (aOR 2.20, 1.82–2.65), sexual violence (aOR 1.77, 1.36–2.31), emotional violence (2.02. 1.61–2.53), physical violence (aOR 1.85, 1.54–2.24), witnessing interparental violence (aOR 1.71, 1.46–2.01), and witnessing community violence (aOR 1.52, 1.26–1.84). Males with disabilities had higher odds of having transactional sex (aOR 4.30, 1.35–13.73), having recent multiple sex partners (aOR 2.31, 1.13–4.75), experiencing emotional violence (aOR 2.85, 1.39–5.82), and witnessing interparental violence (aOR 1.78, 1.12–2.84). HIV models for males did not converge due to low numbers.

**Conclusion:**

Findings emphasize the importance of inclusion and accessibility for adolescents and young adults with disabilities in prevention and services for violence and HIV. Ending HIV in Lesotho depends on addressing the vulnerabilities that lead to potential infection including violence and ensuring equitable services for all.

**Supplementary Information:**

The online version contains supplementary material available at 10.1007/s44197-023-00184-3.

## What is already known on this topic

 To explore the recent state of evidence on HIV and violence among adolescents and young adults with disabilities, we searched PubMed using the terms “HIV”, “violence”, and “disability”, from January 1, 2000 to May 19, 2023. Despite substantial advances in research on violence and HIV, limited evidence exists in documenting prevalence of disability in low- and middle-income countries and examining vulnerabilities of persons with disabilities to HIV and violence using population-based surveys. Systematic reviews and meta-analyses reflect the fact that most research on disabilities and HIV or violence has focused on adults, primarily adult women, and has been conducted in high-income countries. Adolescents and young adults are a priority population in sub-Saharan Africa because of heightened risk of HIV acquisition and vulnerability to violence. Young persons with disabilities may disproportionately experience these risks. Nonetheless, limited data exist examining HIV among persons with disabilities using population surveys. To the best of our knowledge, no research has examined violence among persons with disabilities using population surveys in sub-Saharan Africa.

## What this study adds

 The study provides unique information on the prevalence of functional disability status from a nationally representative household survey among adolescents and young adults in Lesotho. These data expand understanding of the intersectionality of disability, HIV, and violence in the context of a generalized HIV epidemic by examining associations among disability, HIV, and violence. In this national survey of adolescents and young adults, the data enabled a comprehensive analysis of associations between disability, HIV, and violence. This study also includes an examination of sexual risk behaviours associated with HIV acquisition, including condom use, multiple sex partners, and transactional sex.

## How this study might affect research, practice, or policy

Our results provide targets for HIV prevention and care for adolescents and young adults with disabilities in Lesotho as it aims to achieve epidemic control. Youth-friendly HIV services that are accessible and inclusive of persons with disabilities are an essential component to HIV testing, prevention and care. Integrating violence prevention and high-quality post-violence care can accelerate HIV epidemic control.

Prevalence of Disabilities and Violence and HIV among Adolescents and Young Adults, Lesotho, 2018.

## Introduction

Young persons with disabilities have been historically underrepresented in research on health and violence [1–3]. Limited existing research suggests people with disabilities are at elevated risk for experiencing or witnessing violence and HIV. Persons with disabilities may represent a population at elevated risk of violence and HIV due to increased reliance on caregivers and barriers in communication and understanding of risk [1]. Stigma, structural and economic inequities, and lack of economic independence further increase their vulnerability to HIV, sexual risk behaviours, and violence [2, 3]. Functional disabilities refer to difficulties in performing basic everyday tasks or more complex tasks; limitations in functioning may result from physical or mental impairments [4].

Global estimates suggest that more than 15% of the world population lives with some form of disability [4], and 240 million children have disabilities globally [5]. Nonetheless, significant gaps in data exist for low- and middle-income countries (LMICs); limited and incomplete population estimates of disability prevalence exist for sub-Saharan Africa [6]. LMICs tend to have higher prevalence of disability, higher levels of violence, and fewer support services for persons with disabilities [4, 7]. Thus, fully understanding vulnerability to violence and HIV among persons with disability and informing HIV epidemic control efforts requires population data from LMICs. Countries with generalized HIV epidemics, including many countries in sub-Saharan Africa, are important contexts to study associations among disabilities and violence, to understand disparities and provide opportunities to promote health equity at a population level. Given associations between violence and recent HIV acquisition and lower likelihood of having viral load suppression [8], reaching HIV epidemic control by 2030 and reducing inequities depend in part on understanding intersectionalities of disability, violence, and HIV. All young people have a right to be protected from violence and HIV. Understanding the vulnerabilities to violence and HIV among adolescents and young adults living in countries with generalized HIV epidemics can inform efforts to improve the effectiveness of HIV and violence prevention and response strategies. In addition, this information can address disparities in access to services and promote health equity for all young people.

The limited evidence on disability and HIV suggests that persons with disabilities have higher risk of HIV, and women with disabilities have unique vulnerabilities compared to men with disabilities [1]. Among persons with disabilities, sexual violence appears to significantly contribute to increased risk for HIV [9], underscoring the need to examine both HIV and violence among persons with disabilities. Unique gendered patterns may also exist in the association between disability and HIV. One study from Burundi documented higher HIV among women with disabilities compared to women without disabilities, but no association for men [10]. For women with disabilities, sexual violence mediated the association between disability and HIV. In a study from Cameroon, women with disabilities engaged in more paid sexual relationships than women without disabilities [11]. Although both of these studies focused on disability that emerged in childhood, the samples included adults up to age 49. Data on gendered patterns of violence and HIV among adolescents and young adults are needed. Positive HIV status may also compound vulnerability to violence among women with disabilities [12]. On the whole, research suggests higher risk of HIV among persons with disabilities, with unique patterns of association by sex, and violence may play a role in the associations. Recent data from South Africa indicate that HIV is a leading driver of increased disability prevalence [13]. Gaps in research persist for adolescence and young adulthood, which represent developmental periods at unique risk for new HIV infections as well as onset or increases in certain forms of violence [1, 14].

A 2022 meta-analysis documented higher risk for violence against children with disabilities [7]. Children with disabilities had higher odds of any violence, physical violence, sexual violence, and emotional violence, particularly in economically disadvantaged situations. Recent systematic reviews have also documented higher levels of intimate partner violence against women with disabilities [14], which confirm earlier reviews of all violence among adults with disabilities [15]. Data among younger populations are particularly important, as onset of violence among persons with disabilities often occurs prior to age 18 [3]. Gaps identified by a scoping review of measurement of violence against persons with disabilities included a lack of disaggregation by sex and limited availability of population data, particularly community-living individuals rather than institutionalized populations [2]. The authors strongly recommended inclusion of questions about disability in household population surveys, to ensure full representation and inclusion of individuals with disabilities in research.

The lack of available nationally representative data on disability from countries in sub-Saharan Africa, which bears a disproportionate burden of HIV globally, is a barrier to ensuring accessibility of care and reach of epidemic control efforts [8]. Reliance on studies drawing on institutionalized or care-receiving samples may also overestimate associations between disability and HIV and violence, and does not represent the full spectrum of diversity of persons with disabilities [7].

Lesotho has the second-highest HIV prevalence in the world [16] but has made progress towards ending HIV. Lesotho has achieved UNAIDS targets for testing, treatment, and viral suppression, and estimates suggest that Lesotho has reached epidemic control [17]. Lesotho has also made strides in recognizing and protecting basic rights of people with disabilities [18]. Despite these achievements, persistent inequities remain [19], and focused efforts are needed to identify vulnerable populations and ensure health equity in reaching all individuals with prevention and treatment.

As consistent with health equity and social determinants of health frameworks [20, 21], identifying populations disproportionately affected by health problems like HIV and violence can shed light on health disparities. Further, emphasizing equity is core to achieving health outcomes like elimination of violence and reaching HIV epidemic control. This study aimed to document the prevalence of functional disability among adolescents and young adults in Lesotho and assess the association between disability and HIV, sexual risk behaviours, and violence. This study provides the first-ever national estimates of disability among adolescents and young adults in sub-Saharan Africa and examines vulnerability of young people with disability to HIV and violence, stratified by sex.

## Methods

### Study Design and Participants

The nationally representative, cross-sectional 2018 Lesotho Violence Against Children and Youth Survey (VACS) used a standardized household survey methodology that employed a multistage, geographically clustered design. Youth aged 13–24 years participated in an interviewer-administered questionnaire, with data collection between June and September 2018. VACS Lesotho sampled separate enumeration areas for females and males, using the 2016 census sample frame [22]. In stage one, 197 enumeration areas for females and 43 for males were selected using probability proportional to size sampling. This difference in enumeration areas for females reflected oversampling in high-HIV burden areas. The use of sample weights in all analyses adjusted for differences in sample sizes by sex (for more information on sampling and weights, see the Lesotho VACS final report [23]). During stage two, a list of households in each enumerated area was constructed, and 40 households were randomly selected using equal probability systematic sampling. In stage three, one participant aged 13–24 years was randomly selected in selected households. Sampling was done without replacement. The sample size was 8568 (males = 1467, females = 7101), with an overall response rate of 98.0% for males and 96.2% for females. Participants provided informed verbal consent, with parental permission and child assent for participants under age 18. HIV testing was offered to all participants who did not report a previous positive HIV test result. The Lesotho Ministry of Health, Columbia University Medical Center, and CDC independently reviewed and approved the survey protocol.

In the present study, we used 2018 VACS Lesotho data to estimate the prevalence of functional disabilities among adolescents and young adults ages 13–24 in Lesotho. In addition, we assessed HIV and violence by disability status. To address these objectives, questions related to functional disability status, HIV, violence, and sexual risk behaviours were used. Sections 2.3–2.5 Statistical Analysis include details on the methodology of our approach for this secondary data analysis of 2018 VACS Lesotho data.

### Participant Involvement

Participants and their families were not involved in setting the research question or the outcome measures. Adolescents and youth were involved in providing input for adaptation of the VACS questionnaire through an advisory committee for protocol development. Adolescents and youth also provided recommendations for dissemination of VACS results, interpretation of findings, and developing implications for prevention and programming.

### Procedures

Sex-matched interviewers conducted interviews in a safe and private space and recorded responses electronically on netbooks. Extensive interviewer training and field procedures were included in the protocol to increase disclosure and ensure participant safety and confidentiality [24]. Field staff provided service referrals to participants who experienced recent violence or sexual exploitation or tested positive for HIV, as consistent with the protocol response plan. The VACS questionnaire includes questions selected from survey tools with demonstrated validity and reliability that have been cognitively tested and implemented with youth in sub-Saharan African settings [25, 26]. The questionnaire was professionally translated and back-translated into Sesotho for quality assurance and to ensure consistency. Interviews were conducted in English or Sesotho by bilingual interviewers [23].

### Measures

#### Disability

A modified version of the Washington Group—Short Set of Questions on Disability [27] was used to ascertain functional disability status, based on difficulties in functioning due to problems related to: (1) vision; (2) memory or cognition; (3) walking or mobility; (4) self-care (washing or dressing); (5) independent living (doing chores or errands alone); and (6) communication (Supplementary Table). Functional disability was defined as a response of “some difficulty” or “a lot of difficulty” or “cannot function at all” to any of the functional areas [26].

#### HIV and Sexual Risk Behaviours

HIV status was assessed through rapid test or self-report and proof of treatment and dichotomized as HIV positive (positive rapid test result or known HIV positive) or HIV negative (no positive rapid test result and not known HIV positive; [22]). Sexual risk behaviours were defined as recent no or infrequent condom use (in the past 12 months), recent multiple sex partners (in the past 12 months), lifetime transactional sex, and having age-disparate sex partners (Supplementary Table).

#### Violence

Sexual violence included unwanted sexual touching, attempted forced sex, physically forced sex, or any pressured or coerced sex, by any perpetrator. Physical violence included having been slapped, pushed, shoved, shook, intentionally thrown something at, punched, kicked, whipped, beaten with an object, choked, smothered, intentionally burned, attempted drowning, attacked or threatened with a stick, gun, knife or other weapons. Questions asked about physical violence perpetrated by intimate partners, peers, parents, and other adults in the community. Emotional violence was measured by asking participants if a parent, adult caregiver, or other adult relative ever told them that they were not loved, wished they had never been born, or ever ridiculed or put them down. Witnessing interparental violence included seeing or hearing one’s mother or stepmother being punched, kicked, or beaten up by one’s father or stepfather. Witnessing community violence included seeing anyone get attacked outside of the home and family environment (Supplementary Table).

#### Demographic Characteristics and Covariates

Demographic characteristics included marital status (ever married or cohabited), education (currently enrolled in school), orphan status (lost one or both biological parents before age 18), food insecurity (the household does not have enough money for food), and recent employment (worked for money or other wages in the past 12 months). These characteristics were included as covariates in multivariable logistic regression models.

### Statistical Analysis

Analyses included the entire sample of 13–24-year-olds stratified by sex (7101 females and 1467 males). We analysed the associations between disability status and HIV, sexual risk behaviours, and violence using logistic regression stratified by sex, and built multivariable models controlling for marital status, education, food insecurity, orphanhood, and recent employment. Separate models were conducted for each dependent variable, with functional disability status as the independent variable. Survey weights included in all analyses accounted for the complex survey design and generated nationally representative weighted estimates [22, 23]. Missing values were rare with no variable included in these analyses having more than 5% missing. Therefore, missing values were not imputed, rather, a pairwise deletion approach was used to handle missing values [38]. Estimates with relative standard error [RSE] > 30% reflect unstable estimates; results should be interpreted with caution and were suppressed. All statistical analyses were performed using SAS version 9.4 (SAS Institute Inc., Cary, North Carolina, USA), using the SURVEYFREQ and SURVEYLOGISTIC procedures and NOMCAR options to generate robust standard errors that accounted for missing values.

### Role of the Funding Source

Funding for 2018 Lesotho VACS data collection was supported by the U.S. President’s Emergency Plan for AIDS Relief (PEPFAR). This secondary analysis of Lesotho VACS data did not have funding. The funders of the data collection had no role in study design, data collection, data analysis, data interpretation, or writing of the report. All authors except C.R. had full access to all data in the study and the corresponding author had final responsibility for the decision to submit for publication.

## Results

Table 1 includes the prevalence of functional disability, which was significantly higher among females (14̿.1%, 95% CI 12.7–15.4) than males (7.3%, 5.3–9.2; *p* < 0.001). Females had statistically significantly higher prevalence of functional disabilities in vision (6.5% versus 3.6%; *p* = 0.003), cognition (6.4% versus 2.7%; *p* < 0.001), independent living (1.4%; *p* = 0.002), and communication (2.1%; *p* = 0.013). Among females, 11.2% (10.1–12.4) had one disability type. Among males, 6.3% (4.2–8.3) had one disability type. Table 2 includes demographic characteristics by functional disability status among females and males as well as estimates of HIV and any violence by disability status. Among females with disabilities, 10.59% (7.08–14.11) were HIV positive and 66.59% (63.20–70.70) had ever experienced violence. Among females without disabilities, 6.74% (5.90–7.58) were HIV positive and 48.50% (45.61–51.39) had ever experienced violence. Among males with disabilities, 64.65% (52.72–76.57) ever experienced violence. Among males without disabilities, 63.46% (58.80–68.13) ever experienced violence. A significantly lower proportion of females with disability were not enrolled in school (36.5%, 32.3–40.6) compared to females without a disability (43.3%, 41.0–45.6; *p* < 0.001). The difference for males was not statistically significant. Females and males with a disability did not differ significantly from females and males without a disability on marital status, orphan status, food insecurity, or recent employment.

**Table 1:** Prevalence of any functional disability and prevalence of functional disabilities by type among adolescent and young adult females and males aged 13–24 years in Lesotho (2018)

Functional disability type	Females	Males	Chi-squared *p* value
	Weighted % (95% CI)	Weighted % (95% CI)	
	*n* = 7097	*n* = 1467	
Any disability*	**14.1** (**12.7**–**15.4**)	**7.3** (**5.3**–**9.2**)	** < 0.001**
Vision	**6.5** (**5.6**–**7.4**)	**3.6** (**2.1**–**5.1**)	**0.003**
Cognition	**6.4** (**5.6**–**7.2**)	**2.7** (**1.4**–**4.0**)	** < 0.001**
Mobility	0.9 (0.6–1.2)	**	0.922
Self-care	0.6 (0.4–0.8)	**	0.876
Independent living	**1.4** (**1.0**–**1.9**)	**	**0.002**
Communication	**2.1** (**1.7**–**2.6**)	**1.0** (**0.5**–**1.5**)	**0.013**
Number of functional disability types			
None	**85.9** (**84.6**–**87.3**)	**92.7** (**90.8**–**94.7**)	** < 0.001**
1	**11.2** (**10.1**–**12.4**)	**6.3** (**4.2**–**8.3**)	** < 0.001**
2 or more	**2.8** (**2.2**–**3.4**)	**	**0.001**

Bold denotes statistically significant differences between females and males (*p* < 0.05)

*Any disability refers to having one or more functional disability in any domain (vision, cognition, mobility, self-care, independent living, and communication)

**Unstable estimate [relative standard error (RSE) is > 30%]; result should be interpreted with caution

**Table 2:** Demographic characteristics among adolescents and young adults aged 13–24 years, by functional disability status and sex in Lesotho (2018)

	Females			Males		
	Any disability^a^	No disability	Chi-squared *p* value	Any disability	No disability	Chi-squared *p* value
	Weighted % (95% CI)	Weighted % (95% CI)		Weighted % (95% CI)	Weighted % (95% CI)	
Married or cohabiting	*n* = 987	*n* = 6104	0.060	*n* = 102	*n* = 1365	0.977
	21.2 (17.9–24.6)	24.1 (22.1–26.1)		5.9 (0.0–12.2)	5.8 (4.2–7.4)	
Not enrolled in school	*n* = 976	*n* = 6024	** < 0.001**	*n* = 100	*n* = 1322	0.170
	**36.5** (**32.3**–**40.6**)	**43.3** (**41.0**–**45.6**)		32.3 (19.3–45.3)	41.4 (35.3–47.6)	
Orphan^b^	*n* = 960	*n* = 5872	0.973	*n* = 98	*n* = 1339	0.868
	47.7 (44.1–51.2)	47.6 (45.8–49.4)		47.8 (35.6–60.0)	48.7 (44.7–52.8)	
Experienced food insecurity^c^	*n* = 968	*n* = 6038	0.090	*n* = 100	*n* = 1351	0.385
	62.8 (58.5–67.0)	66.4 (64.2–68.6)		63.3 (48.0–78.6)	69.1 (64.2–74.0)	
Recently employed^d^	*n* = 987	*n* = 6102	0.815	*n* = 102	*n* = 1364	0.385
	15.6 (12.4–18.7)	16.0 (14.4–17.5)		28.5 (17.2–39.7)	24.1 (21.3–26.9)	
HIV positive	*n* = 911	*n* = 5648		*n* = 92	*n* = 1284	
	10.59 (7.08–14.11)	6.74 (5.90–7.58)		–	2.42 (1.43–3.41)	
Experienced any violence	*n* = 987	*n* = 6110		*n* = 102	*n* = 1365	
	66.95 (63.20–70.70)	48.50 (45.61–51.39)		64.65 (52.72–76.57)	63.46 (58.80–68.13)	

Bold denotes statistically significant differences between adolescents and young adults with disabilities compared to those without disabilities (*p* < 0.05). – indicates that there were too few males with disabilities who were HIV positive to report an estimate

^a^Any disability refers to having one or more functional disability in any domain (vision, cognition, mobility, self-care, independent living, and communication)

^b^Orphan refers to individuals whose biological mother or father died before age 18

^c^Food insecurity refers to individuals who reported their household did not have enough money for food

^d^Recently employed refers to individuals who worked for money or other payment in the past 12 months

Table 3 includes results of logistic regression analyses. In the univariable model, females who had any functional disability had 1.64 greater odds (1.15–2.35) of being HIV positive and 1.82 greater odds (1.11–2.97) of lifetime transactional sex compared to females with no functional disability. In the multivariable models, HIV infection (adjusted odds ratio [aOR] 1.92, 1.34–2.76) and lifetime transactional sex (aOR 1.79, 1.09–2.95) continued to be associated with disability status. Due to small numbers, models assessing the association between disability and HIV did not converge for males. Univariable models for males identified significant associations between disability status and recent no or infrequent condom use (OR 2.10, 1.10–4.03) and lifetime transactional sex (OR 4.01, 1.10–14.56). Males with any functional disability had 2.23 greater odds (1.18–4.23) of having one or more sexual risk behaviours and 3.24 greater odds (1.3–6.41) of having two or more sexual risk behaviours compared to males with no functional disability. In the multivariable models controlling for demographic covariates, the effect for recent no or infrequent condom use was no longer significant, but disability continued to be associated with lifetime transactional sex (aOR 4.30, 1.35–13.73; *p* = 0.014). The association between disability and recent multiple sex partners was not significant in the univariable model, but the association with disability was significant when controlling for covariates (aOR 2.31, 1.13–4.75, *p* = 0.023). Having a functional disability was associated with engaging in one or more sexual risk behaviours (aOR 2.31, 1.22–4.58, *p* = 0.011) and two or more sexual risk behaviours (aOR 3.42, 1.75–6.71, *p* < 0.001). Having age-disparate sex partners was not significantly associated with disability for males.

**Table 3:** Associations between functional disability status and HIV, sexual risk behaviours, and violence among female and male adolescents and young adults aged 13–24 years in Lesotho (2018)

Females	OR (95% CI)	*p* value	aOR (95% CI)	*p* value
HIV positive^a^	**1.64** (**1.15**–**2.35**)	**0.0072**	**1.92** (**1.34**–**2.76**)	**0.0004**
Sexual risk behaviours				
Recent no or infrequent condom use^b^	0.95 (0.71–1.27)	0.711	0.91 (0.66–1.25)	0.548
Recent multiple sex partners^c^	0.89 (0.60–1.31)	0.545	0.83 (0.55–1.25)	0.361
Lifetime transactional sex^d^	**1.82** (**1.11**–**2.97**)	**0.017**	**1.79** (**1.09**–**2.95**)	**0.023**
Age-disparate sex partner^e^	0.91 (− 0.73 to 1.14)	0.411	0.94 (0.73–1.21)	0.638
One or more sexual risk behaviours	0.95 (0.73–1.24)	0.715	0.98 (0.75–1.30)	0.898
Two or more sexual risk behaviours	0.91 (0.67–1.25)	0.555	0.82 (0.58–1.15)	0.251
Any violence	**2.15** (**1.78**–**2.60**)	** < 0.001**	**2.20** (**1.82**–**2.65**)	** < 0.001**
Sexual violence	**1.63** (**1.25**–**2.11**)	** < 0.001**	**1.77** (**1.36**–**2.31**)	** < 0.001**
Emotional violence	**2.08** (**1.67**–**2.58**)	** < 0.001**	**2.02** (**1.61**–**2.53**)	** < 0.001**
Physical violence	**1.85** (**1.53**–**2.23**)	** < 0.001**	**1.85** (**1.54**–**2.24**)	** < 0.001**
Witnessed interparental violence	**1.65** (**1.40**–**1.95**)	** < 0.001**	**1.71** (**1.46**–**2.01**)	** < 0.001**
Witnessed community violence	**1.55** (**1.29**–**1.88**)	** < 0.001**	**1.52** (**1.26**–**1.84**)	** < 0.001**

Males	OR (95% CI)	*p-*value	aOR (95% CI)	*p-*value
HIV positive	–	–	–	–
Sexual risk behaviours				
Recent no or infrequent condom use	**2.10** (**1.10**–**4.03**)	**0.026**	2.00 (0.97–4.12)	0.061
Recent multiple sex partners	2.00 (0.96–4.10)	0.063	**2.31** (**1.13**–**4.75**)	**0.023**
Lifetime transactional sex	**4.01** (**1.10**–**14.56**)	**0.035**	**4.30** (**1.35**–**13.73**)	**0.014**
Age-disparate sex partner	2.70 (0.63–11.51)	0.178	2.58 (0.50–13.50)	0.260
One or more sexual risk behaviours	**2.23** (**1.18**–**4.23**)	**0.014**	**2.37** (**1.22**–**4.58**)	**0.011**
Two or more sexual risk behaviours	**3.24** (**1.63**–**6.41**)	** < 0.001**	**3.42** (**1.75**–**6.71**)	** < 0.001**
Any violence	1.05 (0.64–1.74)	0.841	1.10 (0.65–1.85)	0.723
Sexual violence	0.29 (0.04–2.00)	0.207	0.31 (0.04–2.15)	0.234
Emotional violence	**2.77** (**1.37**–**5.62**)	**0.005**	**2.85** (**1.39**–**5.82**)	**0.004**
Physical violence	0.93 (0.57–1.52)	0.773	0.96 (0.58–1.59)	0.869
Witnessed interparental violence	**1.78** (**1.16**–**2.72**)	**0.008**	**1.78** (**1.12**–**2.84**)	**0.016**
Witnessed community violence	1.44 (0.85–2.43)	0.175	1.41 (0.84–2.36)	0.194

Separate models were conducted for each dependent variable, with functional disability status as the independent variable. Adjusted models controlled for marital status, education, food insecurity, orphanhood, and recent employment

Bold denotes statistically significant differences between adolescents and young adults with disabilities compared to those without disabilities (*p* < 0.05). The reference group for violence analyses is not experiencing that type of violence

OR, odds ratio; aOR, adjusted OR

^a^HIV positive refers to individuals who had a positive rapid test result or self-reported a recent positive HIV test

^b^Recent no or infrequent condom use refers to individuals who never or infrequently used condoms during sex in the past 12 months; among those who ever had sex

^c^Recent multiple sex partners refers to individuals who had two or more sex partners in the past 12 months; among those who ever had sex

^d^Lifetime transactional sex refers to exchanging sex for money or other goods

^e^Age-disparate sex partner refers to the first sexual partner or one of the three most recent sex partners in the past 12 months being 5 or more years younger or older

– Indicates that the model did not converge

Females with a disability had 2.15 higher odds (1.78–2.60) of having experienced any violence compared with females with no disability. This association remained significant in multivariable models (aOR 2.20, 1.82–2.65). In univariable models, disability status was significantly associated with sexual, physical, and emotional violence, as well as witnessing interparental violence and witnessing community violence. Sexual violence (aOR 1.77, 1.36–2.31), emotional violence (aOR 2.02, 1.61–2.53), and physical violence (aOR 1.85, 1.54–2.24) were all significantly associated with disability in the multivariable models. In multivariable models, females with a disability had significantly higher odds of witnessing interparental violence (aOR 1.71, 1.46–2.01) and witnessing community violence (aOR, 1.52, 1.26–1.84). For males, univariable models revealed significant associations between disability status and emotional violence and witnessing interparental violence, but not any violence, sexual violence, physical violence, or witnessing community violence. In multivariable models, males with a disability had 2.85 higher odds of emotional violence (1.39–5.82) and 1.78 higher odds of witnessing interparental violence (1.12–2.84) than males with no disability.

## Discussion

Using nationally representative population data, we found a substantial proportion of adolescents and young adults in Lesotho have some self-reported functional disability: 1 in 7 females and 1 in 14 males. Critically, findings indicate that young people with functional disabilities in Lesotho have higher odds of violence and HIV, as well as some sexual risk behaviours. Limited data exist on the prevalence of disability among youth from LMICs. This study adds to the body of knowledge on the vulnerabilities of youth with disabilities to violence and HIV. As consistent with the health equity framework [20], the present study emphasizes health inequities underpinning the risks for violence and HIV among adolescents and young adults with disabilities.

Consistent with prior data on prevalence of disability globally and in the sub-Saharan African region [4], adolescent girls and young women had higher prevalence of disability than adolescent boys and young men. This disparity mirrors international findings but is more pronounced. These disparities likely reflect unequal levels of protections and rights among persons with disabilities as well as gender inequities [12, 14]. Families might choose to divert limited financial resources to supporting and prioritizing the health and wellness of male children over that of female children [29]. Higher prevalence of disabilities among females could also reflect disability due to pregnancy and childbirth and limited access to adequate medical care, or disability due to higher rates of domestic violence among women [30]. Differences in prevalence of disabilities between females and males may also indicate reluctance among males to self-disclose functional limitations and reflect stigma related to disabilities and gender norms related to masculinity [2].

Females with functional disabilities had higher odds of HIV infection. However, transactional sex was the only sexual risk behaviour associated with disability among females, suggesting that most risky sexual behaviours such as age-disparate sexual relationships, low condom use, and multiple sex partners do not explain the association between disability status and HIV. The limited sample size for males did not allow for analysis of the association between disability status and HIV. Nonetheless, disability was associated with low condom use in unadjusted analyses and multiple sex partners and transactional sex after controlling for covariates. These findings warrant further research using larger samples for males to fully examine HIV risk among boys and young men with disabilities. Furthermore, the patterns of findings for females and males suggest differences in associations between functional disability and HIV.

Youth with disabilities face barriers to accessing health services and preventive interventions, particularly in low-income countries like Lesotho. The present findings highlight the need for HIV prevention efforts that prioritize outreach for youth with disabilities and support expanded access to services for young people with functional impairments. Adolescent girls and young women in sub-Saharan Africa face complex social, biological, and environmental challenges to avoid HIV infection [31]. They account for a disproportionate number of new HIV infections in sub-Saharan Africa: in Lesotho, they represent less than 10% of the population but 31% of new infections, indicating substantial increased risk compared to other demographic groups [19, 32]. The associations between HIV and disability among adolescent girls and young women in Lesotho points to the need for continued commitment to HIV prevention and treatment through a focus on health equity. Continued focus on adolescent girls and young women is essential to HIV epidemic control, as consistent with PEPFAR’s emphasis on health equity and addressing populations disproportionately affected by HIV. HIV prevention and treatment efforts designed to reach AGYW with disabilities and address intersectionalities of disability and violence can help achieve health equity. In particular, prevention, testing, and treatment services that are accessible for all AGYW can reach young people with disabilities. These services can also incorporate violence prevention components and clinical services for victims of violence.

The association between disability status and lifetime transactional sex was particularly concerning, given the high risk of HIV infection associated with exchanging sex for money or gifts. Youth who experience violence as well as persons with disabilities have increased social and economic vulnerabilities, potentially increasing risk for violence and HIV. Although research on transactional sex has traditionally focused on young women [33, 34], this study found significant associations between disability status and transactional sex for both males and females. These findings emphasize the need for economic opportunities for both male and female adolescents and young adults with disabilities, including educational subsidies to help keep young people in schools and economic empowerment interventions such as cash transfers [35, 36]. Education is a powerful tool for women's equality, particularly in LMICs countries. Initiatives to promote gender equity in education and address inequities among disproportionately affected populations—such as young people with disabilities—can support adolescents and young adults in staying in school and achieving the associated economic benefits. Economic disparity is an ongoing and complex driver of HIV. The DREAMS initiative [35] includes evidence-informed interventions that aim to address many of the risk factors for HIV and violence, such as community mobilization and norms change, social asset building, and social protection. Interventions that address the social and structural drivers of gender inequities can promote improved educational, economic, and health outcomes for all adolescents and young adults. Results of the present study underscore the importance of particular attention on ensuring access and reach for young people with disabilities. The DREAMS model utilizes a multi-sectoral approach of implementing layered evidence-based interventions to reduce vulnerability to HIV and to increase agency among youth. Given the focus on empowerment and sexual risk reduction, DREAMS could reduce transactional sex and its associated risks, though this has not been evaluated.

Findings indicating higher school enrolment among females with disabilities bear further exploration. Lesotho’s Inclusive Education Policy seeks to ensure equity in educational access and inclusion for all learners, including young persons with disabilities [37]. Policies that establish the rights of persons with disabilities to inclusion allow for them to take advantage of the benefits of education and promote equitable access.

Prior to this study, there were gaps in understanding of factors associated with vulnerability for violence and HIV among adolescents and young adults with disabilities in Lesotho. Though some research had explored violence experiences among persons with disabilities, most studies had not included measurement of emotional violence. The present study documented significant associations between functional disability and emotional violence, for both females and males, indicating the need to include emotional violence in studies on disabilities and violence. This study also filled gaps by utilizing population data and examining multiple forms of violence as well as HIV and sexual risk behaviours for people with disabilities. People with disabilities often face barriers to accessing health services and preventive interventions that can strengthen protections against violence and HIV. When services are available, they are often not accessible or inclusive for people with disabilities. HIV prevention efforts that incorporate outreach and strategies that are inclusive and accessible for all adolescents and young adults can address the needs of these highly vulnerable populations and promote health equity in prevention and care. Comprehensive sexual and reproductive health services including access to condoms, education and support for reducing the number of sexual partners, sexually transmitted infection testing and treatment, Pre-Exposure Prophylaxis (PrEP), and family planning are critical tools for prevention and harm reduction among adolescents and young adults.

The findings in this report are subject to at least five limitations. First, this was a cross-sectional study and causality or temporal sequencing of variables of interest cannot be established. Second, self-reported data may be affected by recall bias. Third, limited disclosure might have occurred because of the sensitive nature of the survey, particularly questions asking about violence and disability. Fourth, persons not living in households (e.g., street children, children living in institutions, or students living in dormitories) were not included, and, therefore, these findings reflect the population of adolescents and young adults with functional disabilities living in households. It does not include the experiences of persons with disabilities living in institutionalized settings or those who are unhoused and others with severe functional impairments that precluded them from participating in the interview. Fifth, use of multiple comparisons could increase risk of falsely rejecting the null hypothesis. Finally, the present analyses were not stratified by disability type, due to small numbers.

## Conclusion

Ending HIV in Lesotho will partly hinge on addressing structural vulnerabilities such as inequitable gender norms, violence against women and girls, and equitable access to economic opportunities and health services for persons with disabilities. The results of the present study underscore the important role that inclusion and accessibility for adolescents and young adults with disabilities play for effective HIV and violence prevention and services. These findings demonstrate the need for support and services for persons with disabilities in LMICs, particularly in countries like Lesotho with persistent generalized HIV epidemics. Policies and programming that improve access to health services and other preventive interventions can strengthen protections against violence and HIV for all youth in LMICs.

### Supplementary Information

Below is the link to the electronic supplementary material.

Supplementary file 1 (DOCX 48 kb)

## Data Availability

Deidentified data from the 2018 Lesotho Violence Against Children and Youth Survey are available by request from Together for Girls (https://www.togetherforgirls.org/en/analyzing-public-vacs-data). Additional information is also available, including the Lesotho VACS questionnaires and data codebooks.

## Abbreviations

aOR: Adjusted odds ratio
CDC: Centers for Disease Control and Prevention
CI: Confidence interval
HIV: Human immunodeficiency virus
LMIC: Low- and middle-income countries
OR: Odds ratio
PEPFAR: President’s Emergency Plan for AIDS Relief
RSE: Relative standard error
VACS: Violence Against Children and Youth Survey
