# Supplementary material for: Prevalence of Functional Disabilities and Associations Among Disabilities, Violence, and HIV Among Adolescents and Young Adults in Lesotho
Source: J Epidemiol Glob Health. 2024 Mar 18;14(1):223–33. doi: 10.1007/s44197-023-00184-3 (PMC11043310; doi:10.1007/s44197-023-00184-3)
Supplement: Supplementary file 1 — (DOCX 48 kb) [file 44197_2023_184_MOESM1_ESM.docx]

**Supplementary Table**

**Construction of study variables**

| Variables | Measures |
| --- | --- |
| Functional disabilities* | 1. [Vision] Do you have a serious difficulty seeing, even when wearing glasses, or are you blind? 2. [Memory or cognition] Because of a physical, mental, or emotional condition, do you have serious difficulty concentrating, remembering, or making decisions? 3. [Walking or mobility] Do you have serious difficulty walking or climbing stairs? 4. [Self-care] Do you have difficulty dressing or bathing? 5. [Independent living] Because of a physical, mental, or emotional condition, do you have difficulty doing errands alone such as collecting firewood, collecting water, or shopping? 6. [Communication] Using your usual (customary) language, do you have difficulty communicating, for example understanding or being understood?   Answer options: cannot do at all; a lot of difficulty; some difficulty; no difficulty  Each disability type was coded based on responses of “cannot do at all” or “a lot of difficulty”. Any functional disability included a “yes” response to one or more of the disability questions. |
| HIV status | 1. When was the most recent time you were tested for HIV: less than 3 months ago, 3-6 months ago, 6-12 months ago, or more than 12 months ago? 2. What was the result of that HIV test?   Answer options: HIV positive; HIV negative; uncertain/indeterminate; did not receive test result.  Participants who did not self-report a prior positive HIV test were offered an HIV rapid test, following WHO and national guidelines^†^. Among all participants ages 13-24, 385 self-reported a positive test result, and 7,568 consented to HIV testing, and 7,556 completed testing.  HIV positive was coded based on either self-report of a prior positive HIV test or a positive result on the HIV rapid test. |
| Recent multiple sex partners | How many people have you had sex with in the past 12 months?  Recent multiple sex partners was based on responses of 2 or more. |
| Recent no or infrequent condom use | *Asked of participants who reported they had sex in the past 12 months, for up to 3 sex partners in the past 12 months*.  In the past 12 months when you had sex with this person, how often did you use a condom?  Answer options: always, sometimes, never  Recent no or infrequent condom use was coded based on responding “sometimes” or “never” to wearing condoms during sex for any of their sex partners in the past 12 months. |
| Lifetime transactional sex | *Participants who had sex in the last 12 months:*   1. In the past 12 months, did you ever have sex with this person mainly in order to get things that you need such as money, gifts, or other things that are important to you? 2. Other than the person/people we have already discussed, have you ever had sex with someone mainly in order to get things that you need such as money, gifts, or other things that are important to you?   *Participants who did not have sex in the past 12 months:*   1. Have you ever had sex with someone mainly in order to get things that you need such as money, gifts, or other things that are important to you?   Answer options: yes or no  Lifetime transactional sex was coded based on a “yes” response to any of the transactional sex questions. |
| Having age-disparate sex partner | *Participants were asked of their first sexual partner, and each sexual partner in the past 12 months (up to 3 partners)*  How old was this person? Please give your best guess.  Age-disparate sex partner was coded based on having had one or more sexual partners who were 5 or more years older or younger than the participant at the time of sexual activity. |
| Sexual Violence | Has anyone ever:   1. Touched you in a sexual way without your permission, but did not try and force you to have sex? 2. Make you have sex against your will but did not succeed? 3. Physically forced you to have sex against your will and did succeed? 4. Pressured you in a non-physical way to have sex against your will and did succeed?   Answer options: yes or no  Sexual violence was coded based on a “yes” response to one or more of the sexual violence questions. |
| Emotional violence | Has a parent, adult caregiver or other adult relative ever:   1. Told you that you were not loved, or did not deserve to be loved? 2. Said they wished you had never been born or were dead? 3. Ridiculed you or put you down, for example said that you were stupid or useless?   Answer options: yes or no  Emotional violence was coded based on a “yes” response to one or more of the emotional violence questions. |
| Physical violence | Has a parent, adult caregiver, or other adult relative ever:   1. Slapped, pushed, shoved, shook, pulled hair, twisted arm, pinched, or intentionally threw something at you to hurt you? 2. Punched, kicked, whipped, or beat you with an object? 3. Choked, smothered, tried to drown you, or burned you intentionally? 4. Used or threatened you with a knife, gun or other weapon?   The 4 questions above were repeated for current or former intimate partners, peers, and adults in the community (such as teachers, police, or employers).  Answer options: yes or no  Physical violence was coded based on a “yes” response to one or more of the physical violence questions, for any perpetrator type. |
| Any violence | Any violence was coded based on having answered “yes” to any of the sexual violence, emotional violence, or physical violence questions. |
| Witnessing interparental violence | *For participants ages 13-17:* At any time in your life,  *For participants ages 18-24:* Before the age of 18,  How many times did you see or hear your mother or stepmother being hit, punched, kicked or beaten by your father or stepfather?  Answer options: never, once, more than one time  Witnessing interparental violence was coded if a participant responded “once” or “more than once”. |
| Witnessing community violence | Outside of your home and family environment, how many times did you see anyone get attacked?  Answer options: never, once, more than one time  Witnessing community violence was coded if a participant responded “once” or “more than once”. |

*Functional disability questions from a modified version of the Washington Group-Short Set, excluding the question on hearing and including the American Community Survey question on independent living, modified for country-specific appropriate daily errands (<https://www.census.gov/topics/health/disability/guidance/data-collection-acs.html>).

HIV testing procedures utilized Determine tests with Unigold for test confirmation, following Lesotho Ministry of Health National HIV Testing Services Guidelines and WHO guidelines.
